# Efficacy of oxcarbazepine in treating focal epilepsy based on resting-state EEG functional connectivity and power spectral analyses

**DOI:** 10.1186/s42494-026-00270-6

**Published:** 2026-07-22

**Authors:** Shupeng Cheng, Wenkang Li, Jing Ning, Yingfan Wang, Rong Rong, Xiaoshan Wang

**Affiliations:** 1 Department of Neurology, The Affiliated Brain Hospital of Nanjing Medical University, Nanjing Medical University, Nanjing, 210024 China; 2 School of Rehabilitation Medicine, Nanjing Medical University, Nanjing, 210029 China; 3 Department of Neurology, Nanjing Drum Tower Hospital Clinical College of Nanjing Medical University, Nanjing Medical University, Nanjing, 210008 China

**Keywords:** Oxcarbazepine, Focal epilepsy, Resting-state EEG, Functional connectivity, Power spectral density, Response-related features

## Abstract

**Background:**

Objective electroencephalography (EEG)-based biomarkers are needed to assess oxcarbazepine (OXC) response in patients with focal epilepsy. This study aimed to identify resting-state EEG biomarkers associated with oxcarbazepine efficacy in focal epilepsy using power spectral and functional connectivity analyses.

**Methods:**

In this retrospective cohort study, 27 drug-naïve patients with focal epilepsy underwent resting-state EEG before treatment and approximately 1 year after initiating OXC monotherapy. Nineteen 10–20 system electrodes were recorded at 256/512 Hz, and preprocessed (resampling, detrending, 50 Hz notch, 0.5–70 Hz band-pass, independent component analysis [ICA] artifact removal). Relative power spectral density (rPSD) was estimated via Welch’s method (5 s windows, 50% overlap). Functional connectivity (FC) was quantified by amplitude envelope correlation with correction (AEC-C) within canonical bands, yielding 19×19 matrices. Group comparisons used nonparametric tests with Benjamini–Hochberg false discovery rate (FDR) correction for rPSD and network-based statistic (NBS) for FC (5,000 permutations; initial thresholds *t* = 2.787, *P* < 0.01, and *t* = 3.725, *P* < 0.001). Clinical outcomes were classified as seizure-free (SF) or not seizure-free (NSF) at 6–24 months after OXC initiation.

**Results:**

Baseline clinical characteristics did not differ between groups. At the on-treatment follow-up EEG, the NSF group exhibited significantly higher θ-band rPSD at multiple frontal–central electrodes (Fp1, Fp2, F3, F4, C3, C4, F7, F8, Fz, Cz) after FDR correction. FC analysis showed stronger δ-band connectivity in the NSF group across frontal–central nodes at *P* < 0.01; under the stricter threshold (*P* < 0.001), a robust edge between P3 and F7 remained significantly stronger in the NSF group. No significant between-group differences were observed in other frequency bands after correction.

**Conclusions:**

These findings suggest that the response to oxcarbazepine in focal epilepsy is linked to differential regulation of slow-frequency brain networks. Persistent low-frequency synchronization reflects ongoing network instability and reduced treatment efficacy, whereas attenuation of pathological slow-wave activity indicates effective network stabilization. These band-limited spectral and network features are promising EEG features associated with treatment response to OXC.

## Background

Epilepsy is one of the most common neurological disorders worldwide, characterized by abnormal, excessively synchronized discharges of neuronal populations in the brain that lead to recurrent, transient dysfunction of the central nervous system. Focal epilepsy is the predominant seizure type, accounting for approximately 60% of all patients with epilepsy, and it profoundly affects quality of life, cognitive function, and psychosocial health [1].

Anti-seizure medications (ASMs) are currently the first-line treatment for focal epilepsy. Oxcarbazepine (OXC), a broad-spectrum and highly effective second-generation ASM with proven efficacy and good tolerability, has been widely used as a first-line monotherapy for focal epilepsy [2]. However, clinical practice indicates that roughly one-third of patients respond poorly to initial monotherapy, fail to achieve complete seizure control, and may ultimately develop drug-resistant epilepsy [3]. This interindividual variability in treatment response not only prolongs patient suffering but also poses a substantial challenge for clinicians in therapeutic decision-making. Therefore, identifying predictive biomarkers of OXC efficacy is of critical clinical importance for achieving precise and individualized treatment of epilepsy [4–6].

Electroencephalography (EEG), a noninvasive and convenient technique that reflects cortical electrophysiological activity in real time, plays a key role in the diagnosis and classification of epilepsy [7, 8]. Traditional EEG analysis relies primarily on visual inspection of epileptiform discharges; however, this approach struggles to capture the finer, dynamic changes that reflect functional network states in the brain. With advances in signal processing, quantitative resting-state EEG methods [9]—such as power spectral density (PSD) analysis and functional connectivity (FC) analysis—have provided new perspectives for probing the neuro-pathophysiological mechanisms of epilepsy. PSD analysis quantifies the distribution of oscillatory power across frequency bands, while FC analysis reveals patterns of coordinated information transfer between brain regions. These advanced analytical approaches hold promise for uncovering neural circuit features associated with disease states and medication response in epilepsy.

EEG rhythms are typically divided into δ (1–4 Hz), θ (4–8 Hz), α (8–12 Hz), β (12–30 Hz), and γ (> 30 Hz) bands, which reflect partially distinct neurophysiological processes and are differentially affected by epilepsy and ASMs [7, 9, 10]. In epilepsy, abnormal increases in slow-frequency activity (δ/θ) have been linked to cortical dysfunction and heightened network synchronization, and prior studies consistently report altered background power in low-frequency bands [11, 12]. At the network level, epileptiform discharges can be conceptualized as transient transitions into hypersynchronous oscillatory states, and their recurrence can bias large-scale networks toward slow-frequency synchronization and altered coupling patterns. These band-limited oscillations therefore provide a neurophysiologically interpretable window into epileptic network states and medication effects.

Because spectral and connectivity outcomes can vary with analytic methods, multiple approaches have been compared for power spectrum estimation and connectivity inference, each with trade-offs in bias/variance, computational demand, and sensitivity to noise and volume conduction [13–15]. In this retrospective clinical dataset (19-channel routine EEG), we adopted a single, widely used pipeline (Welch-based PSD and leakage-reduced amplitude envelope correlation) to prioritize computational stability, reproducibility, and comparability across studies, while recognizing that multi-method and source-space comparisons remain important future directions.

To date, some studies have attempted to use resting-state EEG features to evaluate ASM efficacy, but research focusing on specific medications (such as oxcarbazepine) remains limited and yields inconsistent conclusions [10, 16]. This study analyzes resting-state EEG data acquired before OXC initiation and during follow-up under OXC monotherapy in treatment-naïve patients with focal epilepsy, systematically comparing differences in power spectra and functional connectivity network characteristics between seizure-free (SF) and not seizure-free (NSF) patients. We hypothesize that treatment response may be associated with neural oscillatory patterns and network connectivity states confined to specific brain regions and frequency bands. Through this work, we aim to identify candidate on-treatment follow-up EEG features associated with OXC response and to provide objective electrophysiological evidence to support treatment assessment.

## Methods

### Data collection

This was a retrospective study. At the initial visit, attending physicians recorded detailed clinical histories, general health status, and seizure characteristics, which were stored in the electronic health records (EHRs) system. The research team classified epilepsy types based on EHR data and screened patients with focal epilepsy who met the following inclusion criteria:

1. No prior exposure to any ASMs before the first visit;
2. Initiation of OXC monotherapy at or after the first visit and continuation for at least 6 months;
3. Availability of complete and retrievable clinical follow-up data after OXC initiation;
4. No concomitant use of other ASMs or neuropsychiatric medications;
5. Completion of long-term EEG recording before treatment initiation.

Exclusion criteria included:

1. Prior use of other ASMs;
2. Concomitant use of other psychiatric medications;
3. Poor medication adherence;
4. Duration of oxcarbazepine use less than 6 months.

A total of 27 patients with focal epilepsy who met the criteria were included. This study was approved by the Ethics Committee of Nanjing Drum Tower Hospital (approval No. 2021-432-02). All procedures adhered to the ethical principles of the Declaration of Helsinki. Data collection and processing followed privacy protection and data security standards.

Based on EHR records from the first follow-up, 6–24 months after OXC initiation (i.e., the interval from medication start to efficacy assessment), patients were divided into two groups: SF: patients with complete seizure control after treatment and no convulsions or similar manifestations in daily life; and NSF: patients who continued to have seizures after treatment and required adjustments in the therapeutic regimen.

### EEG recording and preprocessing

All patients underwent resting-state EEG acquisition before OXC initiation and at follow-up during ongoing OXC monotherapy. The follow-up EEG was obtained after treatment initiation, rather than after treatment completion or medication withdrawal. Nineteen electrodes were placed according to the international 10–20 system, with electrode locations registered to the ICBM152 standard brain template; the sampling rate was 256–512 Hz. During recording, patients remained awake and at rest. 

EEG preprocessing was performed using Brainstorm software (http://neuroimage.usc.edu/brainstorm) as follows: (1) downsampled data acquired at 512 Hz to 256 Hz to unify sampling rates; (2) removed direct current (DC) offset to eliminate baseline drift; (3) applied a 50 Hz notch filter to remove line noise; (4) applied a 0.5–70 Hz band-pass filter to retain the main EEG frequency range; (5) used independent component analysis (ICA) to identify and remove ocular, muscular, and other artifacts; and (6) based on conventional EEG band definitions, band-pass filtered the preprocessed EEG signals to obtain the δ (1–4 Hz), θ (4–8 Hz), α (8–12 Hz), β (12–30 Hz), and γ (30–50 Hz) band signals.

For each subject and each frequency band, a 60-second resting segment without epileptiform discharges was selected for subsequent analyses. Interictal segments were jointly identified by two experienced electrophysiologists in conjunction with video recordings.

### Relative power spectral density analysis

For the preprocessed EEG data (using the same 60-second artifact- and epileptiform-free segment as reference), PSD was computed in Brainstorm. We used the Welch method (5 s window length, 50% overlap) to estimate the PSD for each EEG channel [17, 18]. Relative PSD values were obtained by scaling each band’s PSD by the total power across the full spectrum: Relative PSD(f) = PSD(f)/$\:{\sum\:}_{i}\left[\mathrm{T}\mathrm{o}\mathrm{t}\mathrm{a}\mathrm{l}\:\mathrm{P}\mathrm{S}\mathrm{D}\right({\mathrm{f}}_{i}\left)\right]$, where f_*i*_ denotes the individual frequencies from the absolute PSD. This procedure has been shown to standardize PSD values across brain regions and subjects [19, 20]. Welch's method was chosen for its high computational efficiency, robustness to noise, and extensive use in resting-state EEG, facilitating comparison with prior pharmaco-EEG and epilepsy studies.

### Functional connectivity analysis

Sensor-level connectivity measures are vulnerable to volume conduction and field spread, which can spuriously inflate zero-lag coupling in conventional coherence- or phase-based metrics. To reduce this bias, we employed amplitude envelope correlation with correction (AEC-C), a leakage-reduced measure that is less sensitive to such artifacts. As a measure of amplitude synchronization of neural oscillations, AEC has demonstrated strong reproducibility and stability in resting-state MEG/EEG connectivity studies [20, 21]. Brainstorm’s built-in connectivity module was used to automate the Hilbert transform and correlation computations, and to estimate complete 19 × 19 adjacency matrices for each band in each subject. Node strength was calculated as the sum of AEC-C values between a given electrode and the other 18 electrodes, representing its total functional connectivity within the network Relative node strength was defined as node strength divided by the total node strength of the current frequency band, representing the proportion of a node's connectivity relative to the total connectivity in that band.

**Fig. 1:**
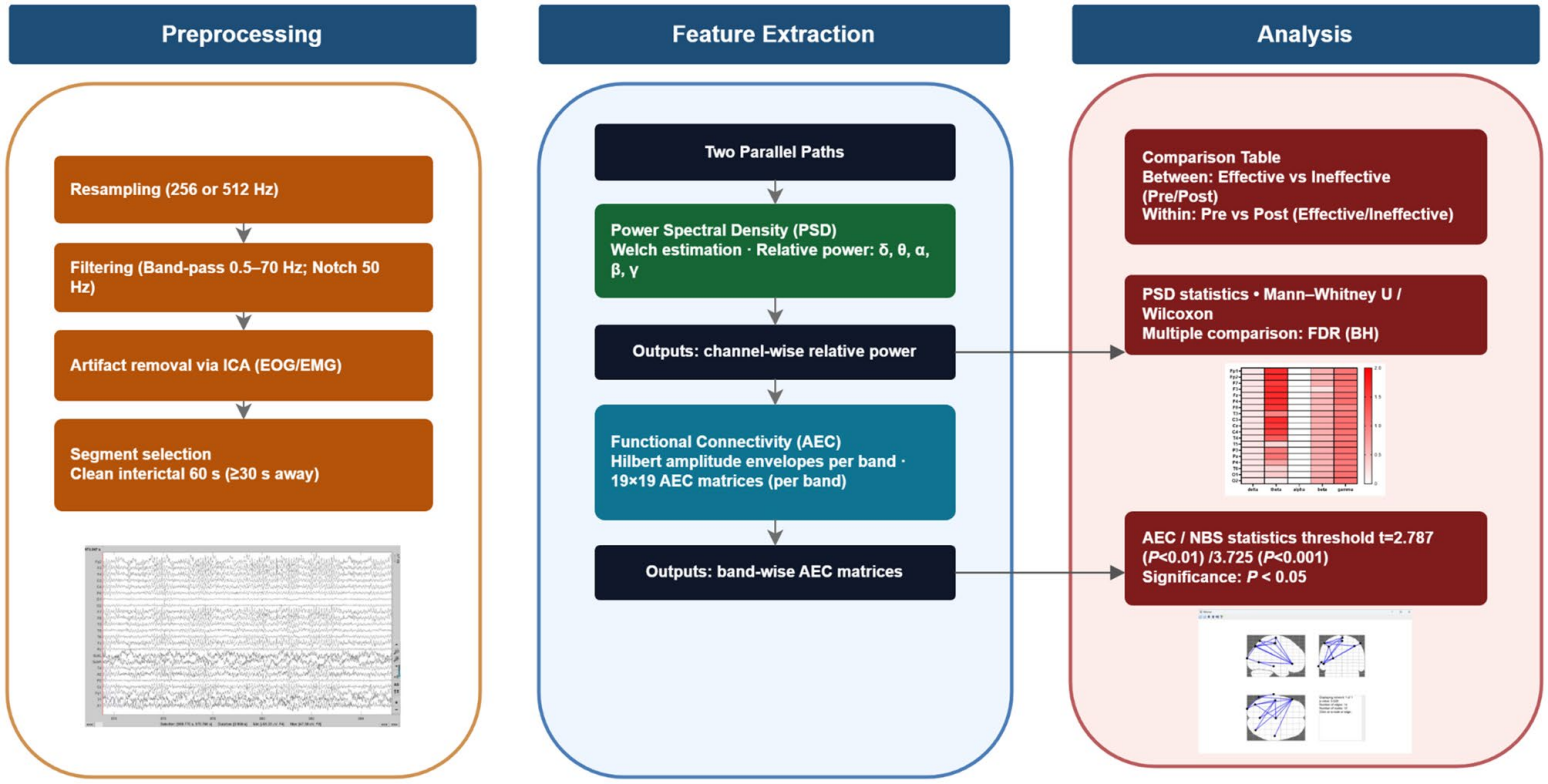
Overview of the process of EEG preprocessing and analysis

The full workflow for EEG data processing and analysis is shown in Fig. 1.

### Statistical analysis

Given the small sample size (fewer than 50 patients), we first used the Shapiro–Wilk test to examine the normality of clinical baseline variables. Fisher’s exact test was used to assess the significance of associations between epilepsy type, MRI findings, comorbidities, and patient outcomes (NSF vs. SF). The Mann–Whitney U test was used to evaluate group differences in mean treatment latency. To evaluate whether differences in follow-up duration could confound group assignment and downstream comparisons, we compared the follow-up time (months) between the SF and NSF groups. Given the small sample size and the non-normal distribution of follow-up duration, a Mann–Whitney U test was used. The relative power spectral density (rPSD) values and AEC-C values were used as metrics for spectral and functional connectivity analyses, respectively. Because both metrics range from 0 to 1 and Shapiro–Wilk tests indicated non-normal distributions, nonparametric methods were used for all analyses.

For functional connectivity, the network-based statistic (NBS) approach was used to perform between-group comparisons of connectivity matrices under different experimental conditions. NBS is a nonparametric network-based method suitable for large-scale multiple-comparison problems inherent in connectivity matrices. It first performs a univariate statistical test on each edge in the matrix, and then, leveraging the topological adjacency among edges, applies cluster-level inference to control the family-wise error rate, thereby improving statistical power while accounting for multiple comparisons [22]. Using the NBS toolbox, we conducted pairwise comparisons of AEC matrices across conditions, with an initial threshold of *t* = 2.787 (*n* = 27, *P* < 0.01) to explore potential large-scale network changes, 5,000 permutations, and a cluster-level significance threshold of *P* < 0.05. To probe more robust local network effects, we also applied a stricter threshold of *t* = 3.725 (*n* = 27, *P* < 0.001) to identify significantly different networks [23]. This dual-threshold strategy follows the official NBS tutorial. Comparisons included: (1) on-treatment follow-up SF vs. NSF, to assess between-group differences in on-treatment follow-up functional connectivity; (2) pre-treatment SF vs. NSF, to compare baseline connectivity differences by eventual treatment outcome; (3) baseline vs. on-treatment follow-up within the SF group, to examine the impact of treatment on connectivity within this subgroup. Only clusters surviving correction at *P* < 0.05 were considered significantly different between conditions.

To evaluate the Group×Time interaction, we implemented NBS within a general linear model (GLM) framework by explicitly modeling Group (SF vs. NSF), Time (baseline vs. follow-up), and their interaction term (Group×Time) in the design matrix. This approach identified subnetworks showing differential longitudinal changes between SF and NSF. Prior to model fitting, connectivity matrices were Fisher r-to-z transformed to improve distributional characteristics for edge-wise statistics; importantly, statistical significance for the interaction effect was still obtained via permutation-based cluster-level inference, thereby avoiding strong parametric assumptions. Inference settings matched those of the NBS analyses above (5,000 permutations, cluster-level *P* < 0.05, dual initial thresholds). For band-limited relative power analyses, nonparametric tests were likewise used for between- and within-group comparisons. For the two independent samples (follow-up SF vs. NSF), the Mann–Whitney U test was applied; for paired comparisons within the SF group (baseline vs. follow-up), the Wilcoxon signed-rank test was used.

To assess the interaction between treatment and clinical response, we additionally performed a change-score analysis by computing ΔrPSD = (follow-up − baseline). Because the distribution of Δ values did not consistently satisfy normality assumptions, ΔrPSD was compared between SF and NSF using the Mann–Whitney U test. With only two time points, this between-group difference in Δ provides an interpretable test of the Group×Time interaction.

To address multiple comparisons across electrodes and frequency bands, all power-spectrum results were corrected using the Benjamini–Hochberg false discovery rate (FDR) procedure. With a prespecified α level, this method controls the proportion of false positives; in this study, the FDR threshold was set to 0.05, i.e., *q* < 0.05 after correction was considered significant. All network statistics were performed in MATLAB (version R2024a; MathWorks, Natick, MA, USA) using the NBS toolbox (version 1.2) and BrainNet Viewer (version 1.7). All statistical analyses were performed using SPSS Statistics (version 29.0; IBM Corp., Armonk, NY, USA), Python (version 3.11.9; package versions to be specified), and GraphPad Prism (version 11.0.0; GraphPad Software, San Diego, CA, USA), and results were cross-checked by the research team to ensure accuracy and reliability.

## Results

### Clinical baseline characteristics

Among the 27 eligible patients (15 in the NSF group and 12 in the SF group), the sex distributions were comparable (NSF: 7 males, 8 females; SF: 6 males, 6 females; no significant difference was observed [*P* = 0.863]). Age (median [IQR]) was 23.0 [19.0–38.5] years in NSF and 20.5 [16.8–27.8] years in SF, with no significant between-group difference (*P* = 0.557). The mean interval from seizure onset to treatment was 23.58 months in NSF, slightly longer than 22.87 months in SF, but not significantly different (*P* = 0.548). Nine patients had temporal lobe epilepsy (33% of total), and seven had MRI abnormalities (26%); neither showed significantly between groups differences (temporal lobe epilepsy *P* = 0.217; MRI abnormalities *P* = 0.408). Follow-up duration (months) was also comparable between the NSF and SF groups (Mann–Whitney U test: *U* = 85.0, *Z* = − 0.245, *P* = 0.806; NSF: *n* = 15, 20.0 [14.0–27.0]; SF: *n* = 12, 22.0 [19.5–25.0]). Overall, no significant between-group differences were observed in baseline characteristics prior to treatment (Table 1). We processed and analyzed interictal data for functional connectivity and spectral power before and after treatment in all 27 patients.

**Table 1:** Baseline characteristics and between-group comparisons

Variable	NSF (*n* = 15)	SF (*n* = 12)	Test statistic	*P* value
Male, *n* (%)	7 (46.67)	6 (50.00)	0.030^a^	0.863
Age, years, median (IQR)	23.0 (19.0, 38.5)	20.5 (16.8, 27.8)	78.000^b^	0.557
Seizure-to-treatment interval, months, mean EEG-to-follow-up interval, months, median (Q1, Q3)	23.58 20.0 (14.0, 27.0)	22.87 22.0 (19.5, 25.0)	77.000^b^ 85.000^b^	0.548 0.806
Temporal lobe epilepsy, *n* (%)	7 (46.67)	2 (16.67)	Fisher	0.217
MRI-negative, *n* (%)	10 (66.67)	10 (83.33)	Fisher	0.408
Any complications, *n* (%)	2 (13.33)	1 (8.33)	Fisher	1.000
Cavernous hemangioma	1 (6.67)	0 (0.00)	-	-
Psychiatric disorder	1 (6.67)	0 (0.00)	-	-
Suspected CNS infection	0 (0.00)	1 (8.33)	-	-

Notes: a χ² test; b Mann–Whitney *U* test;

Temporal lobe epilepsy, MRI-negative status, and complications were compared using Fisher’s exact test;

‘-’ indicates not reported or not applicable. *NSF* Not seizure-free, *SF* Seizure-free

### Spectral power analysis

We first computed rPSD at each electrode before and after treatment in both groups to characterize scalp EEG power distribution within each frequency band. In the delta band, between-group differences were primarily confined to the right parietal electrode (P4). In the alpha band, differences appeared at the midline central electrode (Cz) and right parietal electrode (P4). In the beta band, differences were observed at the right occipital electrode (O2). In the gamma band, differences were most widespread, involving bilateral posterior temporal (T5, T6) and bilateral occipital (O1, O2) electrodes. However, after FDR correction, none of the between-group differences at any channel remained statistically significant.

**Fig. 2:**
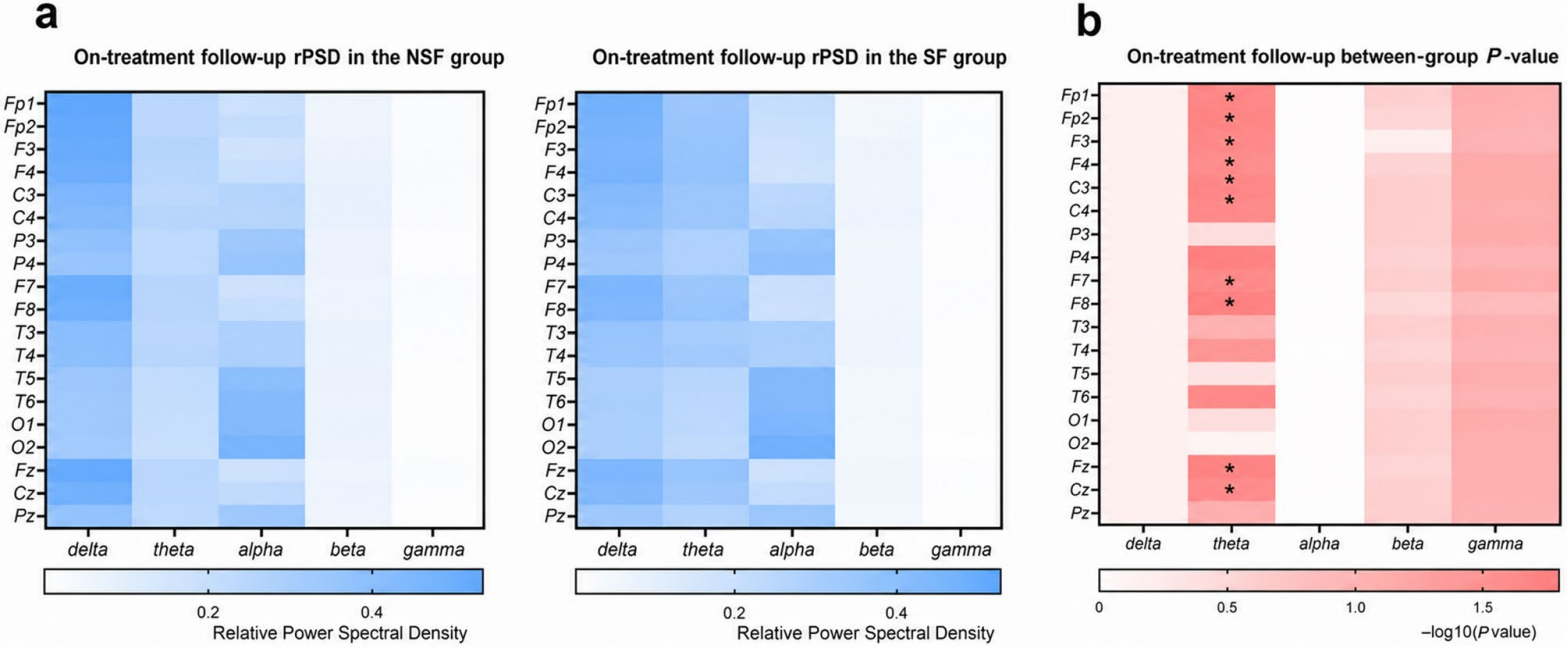
On-treatment follow-up relative power spectral density (rPSD) and between-group differences. (**a**) Heatmaps showing follow-up rPSD during OXC monotherapy across frequency bands for the NSF and SF groups. (**b**) Heatmap of between-group differences in follow-up rPSD, expressed as the −log10(*P* value). *P* value were corrected for multiple comparisons using false discovery rate (FDR) correction. Asterisks indicate statistically significant between-group differences (*FDR-corrected *P* < 0.05)

In the on-treatment follow-up recordings, the most robust group difference emerged in the θ band (4–8 Hz): patients in the NSF group exhibited significantly higher relative θ power than those in the SF group at multiple frontal and central electrodes (Fp1, Fp2, F3, F4, C3, C4, F7, F8, Fz, Cz), and these differences remained statistically significant after FDR correction (Fig. 2). This pattern indicates that seizure freedom after OXC is associated with a relative attenuation of frontal–central slow-frequency oscillatory activity, whereas the NSF group shows persistence of elevated θ power. Importantly, the interaction effect between treatment and clinical response (Group × Time interaction) did not remain significant after correction for multiple comparisons.

### Functional connectivity analysis

**Fig. 3:**
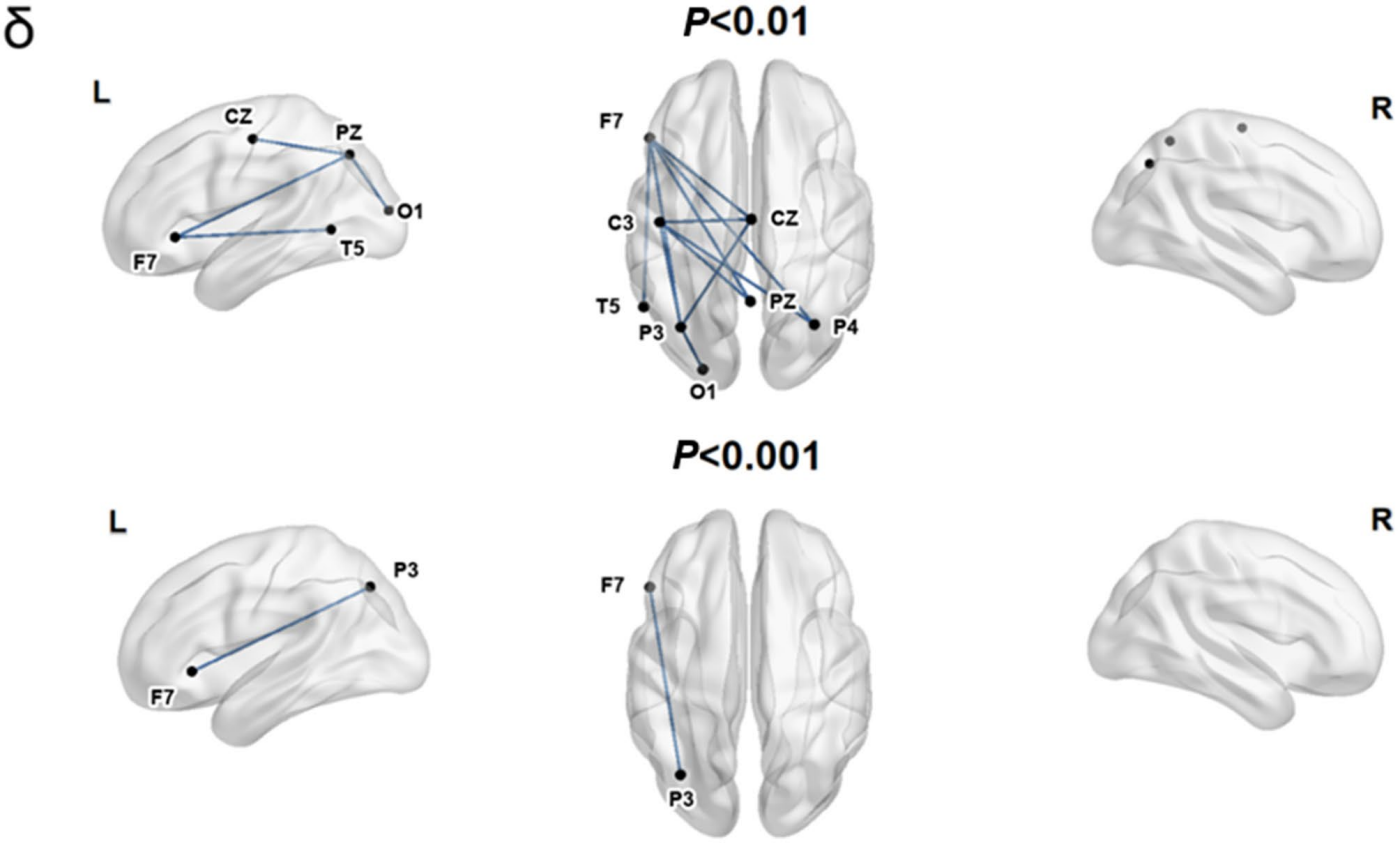
Group differences in functional connectivity in the delta band. Network-based statistics revealed significant between-group differences at *P* < 0.01 (upper panel) and *P* < 0.001 (lower panel). Nodes represent the locations of electrode leads, and edges indicate altered functional connections (L, left; R, right)

Using AEC-C, we constructed functional connectivity matrices between electrodes and conducted nonparametric, NBS across frequency bands to compare connectivity strength between the two groups. As shown in Fig. 3, at a statistical threshold of *P* < 0.01, on-treatment follow-up patients in the NSF group exhibited significantly stronger delta-band connectivity across multiple frontal and central electrodes (including Fp1, Fp2, F3, F4, C3, C4, F7, F8, Fz, and Cz) compared with the SF group. When the statistical threshold was further tightened to *P* < 0.001, a smaller but more robust significant connection was observed: connectivity between the left parietal electrode P3 and the left anterior temporal electrode F7 was significantly stronger in the NSF group. In other frequency bands, no statistically significant between-group differences in connectivity strength were found after correction. The interaction effect for functional connectivity also failed to reach statistical significance.

## Discussion

Based on resting-state EEG analyses integrating FC and PSD, this study explored the neurophysiological underpinnings of OXC in the treatment of focal epilepsy and sought potential biomarkers of its therapeutic efficacy. The key findings reveal that, following OXC treatment, patients in the SF group not only achieved better seizure control but also exhibited marked remodeling of brain functional network patterns and band-specific neural oscillatory activity, with significant differences from the NSF group. These findings provide novel electrophysiological understanding of OXC’s anti-seizure mechanisms and may facilitate the development of individualized epilepsy treatment strategies.

An important finding from the power spectrum analysis offers a new perspective for differentiating treatment responses. At on-treatment follow-up, the θ band rPSD at multiple frontal and central electrodes was significantly higher in non-responders than in responders. Physiologically, θ band activity is linked to various cognitive functions; pathologically, its abnormal elevation is commonly viewed as a marker of cortical dysfunction and is closely associated with epileptic activity [5, 24, 25]. For example, in patients with drug-resistant temporal lobe epilepsy, deficits in executive control are significantly associated with alterations in θ band functional connectivity [25]. This implies that, if OXC effectively improves cognitive function, its mechanism may involve modulating or “normalizing” these abnormal θ band activities. Prior studies have reported enhanced low-frequency power in background EEG activity among epilepsy patients [11, 12], while ASMs attenuate it [26–28]. Jun and colleagues used standardized low-resolution brain electromagnetic tomography (sLORETA) to show OXC significantly decreased background low-frequency EEG activity in the left frontotemporal and limbic regions in children with benign childhood epilepsy with centrotemporal spikes (BCECTS), after six months [16]. In our cohort, we did not observe a statistically significant pre-to-post reduction in low-frequency power across the entire cohort after FDR correction, which may reflect limited sample size and inter-individual variability. Nonetheless, θ band power remained lower in the SF group compared with the NSF group, consistent with the concept that effective therapy can attenuate pathological slowing. Our prior work examined whether EEG microstates could predict response to OXC monotherapy in newly diagnosed focal epilepsy and found specific microstate metrics associated with therapeutic response [5]. Although that study did not directly elucidate how OXC alters microstates or band-limited power, the dynamics of microstates reflect the macroscopic patterns of functional brain network activity, and such alterations may be manifested in EEG spectral characteristics. In the present study, the persistently elevated θ power over frontal and central regions in the NSF group after treatment strongly suggests that OXC fail to effectively correct the pathophysiological state of these key regions. As hubs for executive function, motor control, and higher-order cognitive activities cognition, persistent abnormal oscillatory activity in these regions may constitute the electrophysiological basis for the lack of clinical improvement in the NSF group. Although no prior literature has directly quantified θ band spectral power changes under OXC monotherapy, our findings may help explain the electrophysiological effects associated with OXC treatment. Accordingly, follow-up θ power, particularly over frontal and central regions, may serve as an objective EEG feature associated with treatment response, reflecting whether OXC has modulated functional activity in key cortical regions and providing complementary electrophysiological information for clinical evaluation.

Clinically, the functional connectivity findings showed even stronger discriminatory power. We observed a key phenomenon: the NSF group exhibited significantly stronger delta-band (1–4 Hz) functional connectivity than the SF group, and under the stricter threshold (*P* < 0.001), robust differences were specifically concentrated along the pathway between left parietal P3 and left anterior temporal F7. In the pathophysiology of epilepsy, the abnormal increases in slow-wave activity are closely related to heightened neuronal network synchronization, and such excessive synchrony is considered central to the formation and maintenance of epileptic networks. Experimental neocortical-injury-induced models further suggest that persistent status epilepticus can be sustained by specific, excessively connected recurrent circuits, particularly in slow-frequency bands, which may represent a convergent mechanism of network hyperexcitability across etiologies [29]. As a sodium-channel blocker, OXC stabilizes neuronal membrane potentials and reduces excitability, thereby suppressing the generation and propagation of abnormal discharges at their source [30]. Therefore, the reduction in slow-wave connectivity in the SF group after treatment suggests that OXC successfully attenuated pathological neuronal synchronization. The persistent δ band hyperconnectivity observed in the NSF group—especially its broad distribution across key frontal and central nodes—strongly indicates that OXC failed to dismantle the underlying core network supporting seizure generation and propagation. This finding helps explain why clinical symptoms in these patients were not effectively controlled. Our results provide evidence at the macroscopic brain network level for the pharmacological action of OXC, indicating that it achieves seizure control by “desynchronizing” and weakening or dismantling abnormal functional networks that support epileptic activity. The absence of such changes in the NSF group, conversely, supports the notion that this network remodeling process is a key link in OXC’s therapeutic action. This differential finding has strong translational potential, suggesting that monitoring δ band functional connectivity during treatment may serve as an early response-related features to assist in identifying patients with insufficient treatment response, thereby providing an objective basis for timely regimen adjustments.

### Limitations

This study has several limitations. First, the sample size was relatively small, which may have precluded the detection of subtle yet genuine effects in other frequency bands. Although the main findings withstood multiple-comparison correction, they should still be interpreted with caution, particularly in the absence of validation in an independent cohort. Another limitation is the variability in follow-up duration across patients, which may have led to potential misclassification in a small number of seizure-free patients with shorter follow-up. Future studies should use a more standardized and sufficiently long follow-up period to improve outcome classification. In addition, although all follow-up EEG recordings were obtained during continuous oxcarbazepine monotherapy, differences in treatment duration, medication dose, treatment stage, and the natural disease course may still have influenced EEG-derived measures. These factors should be more carefully controlled in future studies. Finally, we did not perform subgroup analyses according to seizure focus origin. Future studies with larger samples should further investigate the influence of seizure focus location and integrate multimodal imaging or machine learning approaches to improve outcome prediction.

## Conclusions

In summary, through long-term follow-up, this study identified on-treatment follow-up frontal-central theta power and θ band functional connectivity as candidate EEG features associated with response to OXC. The SF group showed relatively attenuated slow-frequency power and connectivity, whereas the NSF group exhibited persistent slow-wave synchronization and hyperconnectivity. Together with prior pharmaco-EEG and source-localization evidence suggesting that OXC modulates low-frequency background activity, these findings improve our understanding of inter-individual variability in electrophysiological responses to OXC and support further investigation of EEG features in treatment evaluation [24, 31].

## Data Availability

De-identified participant data collected in this study will be provided upon reasonable request after signing an appropriate data-sharing agreement. Data access requests should be submitted to the corresponding author. All requests require approval from the appropriate ethics committees and data custodians. Data and related documents will be made available upon publication.

## Abbreviations

AEC-C: Amplitude envelope correlation with correction
ASMs: Antiseizure medications
BCECTS: Benign childhood epilepsy with centrotemporal spikes
EEG: Electroencephalography
EHRs: Electronic health records
FC: Functional connectivity
FDR: False discovery rate
GLM: General linear model
ICA: Independent component analysis
ICBM: International consortium for brain mapping
MEG: Magnetoencephalography
MRI: Magnetic resonance imaging
NBS: Network-based statistic
NSF: Not seizure-free
OXC: Oxcarbazepine
PSD: Power spectral density
rPSD: Relative power spectral density
SF: Seizure-free
sLORETA: Standardized low-resolution brain electromagnetic tomography
STROBE: Strengthening the reporting of observational studies in epidemiology
